# Diameter-sensitive biocompatibility of anodic TiO_2_ nanotubes treated with supercritical CO_2_ fluid

**DOI:** 10.1186/1556-276X-8-150

**Published:** 2013-04-02

**Authors:** Ming-Ying Lan, Chia-Pei Liu, Her-Hsiung Huang, Jeng-Kuei Chang, Sheng-Wei Lee

**Affiliations:** 1 Department of Otolaryngology, Taipei Veterans General Hospital, Taipei, 11217, Taiwan; 2 Institute of Clinical Medicine, National Yang-Ming University, Taipei, 11221, Taiwan; 3 Institute of Materials Science and Engineering, National Central University, No. 300, Jhongda Road, Jhong-Li, Taoyuan, 32001, Taiwan; 4 Department of Dentistry, National Yang-Ming University, Taipei, 11221, Taiwan

**Keywords:** Biocompatibility, TiO_2_ nanotubes, Anodic oxidation, Supercritical CO_2_ fluid, Human fibroblast cells, 68.47.Gh, 82.45.Yz, 82.50.Hp, 87.17.-d

## Abstract

This work reports on the diameter-sensitive biocompatibility of anodic TiO_2_ nanotubes with different nanotube diameters grown by a self-ordering process and subsequently treated with supercritical CO_2_ (ScCO_2_) fluid. We find that highly hydrophilic as-grown TiO_2_ nanotubes become hydrophobic after the ScCO_2_ treatment but can effectively recover their surface wettability under UV light irradiation as a result of photo-oxidation of C-H functional groups formed on the nanotube surface. It is demonstrated that human fibroblast cells show more obvious diameter-specific behavior on the ScCO_2_-treated TiO_2_ nanotubes than on the as-grown ones in the range of diameters of 15 to 100 nm. This result can be attributed to the removal of disordered Ti(OH)_4_ precipitates from the nanotube surface by the ScCO_2_ fluid, thus resulting in purer nanotube topography and stronger diameter dependence of cell activity. Furthermore, for the smallest diameter of 15 nm, ScCO_2_-treated TiO_2_ nanotubes reveal higher biocompatibility than the as-grown sample.

## Background

Titanium (Ti) and its alloys have been widely used for dental and orthopedic implants because of their favorable mechanical properties, superior corrosion resistance, and good biocompatibility [1-3]. When exposed to the atmosphere, the Ti metal spontaneously forms a thin, dense, and protective oxide layer (mainly TiO_2_, approximately 10 nm thick) on its surface, which acts like a ceramic with superior biocompatibility. When the Ti implant is inserted into the human body, the surrounding tissues directly contact the TiO_2_ layer on the implant surface. Therefore, the surface characteristics of the TiO_2_ layer determine the biocompatibility of Ti-based implants. Earlier studies primarily investigated the influence of surface topography of implants on cell behaviors at the micrometer scale [4-6]. Recently, the interaction of nanometric scale surface topography, especially in the sub-100-nm region, with cells has been recognized as an increasingly important factor for tissue acceptance and cell survival [7-9]. Various nanotopography modifications have been proposed to enhance the cell responses to the Ti-based implants. For example, TiO_2_ nanowire scaffolds fabricated by hydrothermal reaction of alkali with the Ti metal, mimicking the natural extracellular matrix in structure, can promote the adhesion and proliferation of mesenchymal stem cells (MSCs) on Ti implants [10]. Chiang et al. also proposed that a TiO_2_ multilayer nanonetwork causes better MSC adhesion and spreading, as well as faster cell proliferation and initial differentiation [11].

In the recent years, self-organized TiO_2_ nanotubes fabricated by electrochemical anodization of pure Ti foils have attracted considerable interest owing to their broad applications in photocatalysis [12], dye-sensitized solar cells [13], and biomedical field [14,15]. A major advantage of anodic oxidation is the feasibility to well control the diameter and shape of the nanotubular arrays to the desired length scale, meeting the demands of a specific application by precisely controlling the anodization parameters. In a number of studies on the cell response to TiO_2_ nanotubes, nanosize effects have been demonstrated for a variety of cells [16-18]. Park et al. reported that vitality, proliferation, migration, and differentiation of MSCs and hematopoietic stem cells, as well as the behavior of osteoblasts and osteoclasts, are strongly influenced by the nanoscale TiO_2_ surface topography with a specific response to nanotube diameters between 15 and 100 nm [19]. Furthermore, even if the surface chemistry of the nanotubes is completely modified with a dense alloy coating onto the original nanotube layers, the nanosize effects still prevail [20]. In other words, the cell vitality has an extremely close relationship with the geometric factors of nanotube openings.

On the other hand, using supercritical CO_2_ (ScCO_2_) as a solvent has shown many advantages when chemically cleaning or modifying the surface of materials. The high diffusivity and low surface tension of ScCO_2_ enable reagents to access the interparticle regions of powders, buried interfaces, or even nanoporous structures that cannot be reached using conventional solution or gaseous treatment methods [21,22]. Recent studies have shown that ScCO_2_ is an effective alternative for terminal sterilization of medical devices [23]. It was also reported that the contact of ScCO_2_ with P25 TiO_2_ (70% anatase and 30% rutile), which contains both anatase and rutile phases, leads to the formation of a variety of functional groups and substantially modifies the surface chemistry [24]. Moreover, the ScCO_2_ drying technique has been proven to effectively reduce intertube contacts and to produce bundle-free and crack-free TiO_2_ nanotube films [25]. The aim of this study is to gain an understanding of the influence of ScCO_2_ on surface topography and chemistry of anodic TiO_2_ nanotubes and also to study the diameter-specific biocompatibility of these ScCO_2_-treated TiO_2_ nanotubes with human fibroblast cells. The human fibroblast cell behavior, including cell adhesion, proliferation, and survival, in response to the diameter of TiO_2_ nanotubes is investigated.

## Methods

### Preparation of ScCO_2_-treated TiO_2_ nanotubes

Self-organized TiO_2_ nanotubes were prepared by electrochemical anodization of Ti foils (thickness of 0.127 mm, 99.7% purity, ECHO Chemical Co. Ltd., Miaoli, Taiwan). A two-electrode electrochemical cell with Ti anode and Pt as counter electrode was used. All anodization experiments were carried out in ethylene glycol electrolytes containing 0.5 wt.% NH_4_F at 20°C for 90 min. All electrolytes were prepared from reagent-grade chemicals and deionized water. Anodization voltages applied were between 10 and 40 V, and resulted in nanotube diameters ranging from 15 up to 100 nm. The TiO_2_ nanotubes with the diameter of 100 nm annealed at 400°C for 2 h were also prepared as the reference sample. After the electrochemical process, the nanotube samples were cleaned ultrasonically with deionized water for 1 h to remove the residual by-products on the surface. Subsequently, ScCO_2_ fluid (99.9% purity) was utilized to treat the nanotubes at the temperature of 53°C and in the pressure of 100 bar for 2 h. For the *in vitro* experiments, low-intensity UV light irradiation (<2 mW/cm^2^) was performed on all nanotube samples using fluorescent black-light bulbs for 8 h.

### Material characterization

Field emission scanning electron microscopy (FE-SEM; FEI Quanta 200 F, FEI, Hillsboro, OR, USA) was employed for the morphological characterization of the TiO_2_ nanotube samples. X-ray diffraction (XRD) was utilized to determine the phase of the TiO_2_ nanotubes. The surface wettability of materials was evaluated by measuring the contact angle between the TiO_2_ nanotubes and water droplets in the dark. Contact angle measurements were performed at room temperature by the extension method, using a horizontal microscope with a protractor eyepiece. In addition, in order to investigate the functional groups possibly formed during the ScCO_2_ process, X-ray photoelectron spectroscopy (XPS) was employed to analyze the carbon spectra (in terms of C 1*s*) on the nanotube surfaces.

### Cell culture

MRC-5 human fibroblasts were received from the Bioresource Collection and Research Center, Taiwan. Cells were plated in a 10-cm tissue culture plate and cultured with Eagle's minimum essential medium (Gibco, Life Technologies Corporation, Grand Island, NY, USA) containing 10% fetal bovine serum, 2 mM l-glutamine, 1.5 g/L sodium bicarbonate, 0.1 mM non-essential amino acids, and 1.0 mM sodium pyruvate. Cultures were maintained at 37°C in a humidified atmosphere of 5% CO_2_. Cells were then seeded onto the autoclaved titanium samples placed in a 12-well culture plate (Falcon, BD Biosciences, San Jose, CA, USA) at a density of 5 × 10^3^ cells/cm^2^ for 3 days for cell adhesion assay and 1 × 10^4^ cells/cm^2^ for 1 week for cell proliferation assay, respectively.

### Cell adhesion

For cell adhesion experiments, 3 days after cell plating, non-adherent cells were washed with phosphate-buffered saline (PBS). The adherent cells were fixed in 4% paraformaldehyde (USB Corp., Cleveland, OH, USA) for 1 h at room temperature and washed with PBS. After fixation, the cells were permeabilized with 0.1% Triton X-100 (Sigma-Aldrich Corporation, St. Louis, MO, USA) in PBS for 15 min at 4°C. Cells were then washed with PBS and incubated with rhodamine phalloidin (Life Technologies Corporation, Grand Island, NY, USA) for 15 min for actin filament stain and with diamidino-2-phenylindole (DAPI; Thermo Fisher Scientific Inc., Waltham, MA, USA) for 5 min for nuclei stain. The images of the stained fibroblasts were taken using a fluorescent microscope to examine the cell adhesion morphology and cytoskeletal arrangement. For SEM observation, cells were fixed with 2.5% glutaraldehyde solution (Merck & Co., Inc., Whitehouse Station, NJ, USA) for 1 h at room temperature. Samples were rinsed in PBS solution twice, dehydrated in a series of ethanol (40%, 50%, 60%, 70%, 80%, 90%, and 100%) and critical point dried with a critical point dryer (CPD 030, Leica Microsystems, Wetzlar, Germany).

### Cell proliferation

Additional cell proliferation was quantified 1 week after cell plating at a density of 1 × 10^4^ cells/cm^2^ using cell proliferation reagent WST-1 (Roche, Woerden, Netherlands) according to the manufacturer's instructions. On the 7th day, cells on the nanotubes were washed with PBS twice. The cells were incubated with a medium containing 10% WST-1 cell proliferation reagent at 37°C in a humidified atmosphere of 5% CO_2_ for 2 h. The solution was then retrieved from each well to a 96-well plate, and optical densities were measured using a spectrophotometer (Tecan Group Ltd., Männedorf, Switzerland) at 450 nm. All experiments were carried out in triplicate, and at least three independent experiments were performed. Data were presented as mean ± standard deviation and analyzed by analysis of variances using SPSS 12.0 software (SPSS Inc., Chicago, IL, USA). A *p* value of <0.05 was considered statistically significant.

## Results and discussion

**Figure 1:**
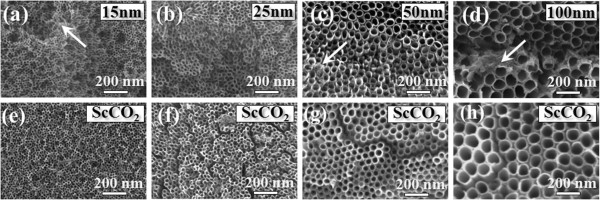
**SEM images of self-organized TiO**_**2 **_**nanotubes with different diameters.** The nanotubes are in the range of 15 to 100 nm before (**a** to **d**) and after (**e** to **h**) the ScCO_2_ treatment. Disordered Ti(OH)_4_ precipitates are indicated by white arrows.

**Figure 2:**
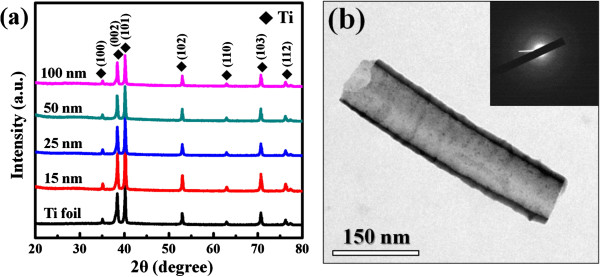
**XRD spectra and TEM image of as-grown TiO**_**2 **_**nanotubes.** (**a**) XRD spectra of as-grown TiO_2_ nanotubes with different diameters and (**b**) TEM image taken from an as-grown nanotube with the diameter of 100 nm. The inset also shows the corresponding diffraction pattern.

Figure 1a,b,c,d shows the SEM micrographs of as-anodized TiO_2_ nanotubes with the diameters of 10, 25, 50, and 100 nm produced by electrochemical anodization at the applied voltages of 5, 10, 20, and 40 V, respectively. The diameters of as-grown nanotubes are nearly proportional to the applied voltages. As shown in the XRD spectra of Figure 2a, only peaks related to the Ti foil are observed, indicating that all as-anodized TiO_2_ nanotubes are mainly amorphous phase, likely to be TiO_2_·*x*H_2_O [26]. Figure 2b shows a representative TEM image taken from an as-grown nanotube with the diameter of 100 nm. The corresponding diffraction pattern reconfirms that the nanotubes are non-crystalline. We also find that even after being cleaned ultrasonically in water for 1 h, the nanotube surface is partially covered by irregularly shaped and disordered structures, as indicated by white arrows. These disordered structures should be Ti(OH)_4_ precipitates formed via the instantaneous hydrolysis reaction, which leads to the generation and accumulation of Ti(OH)_4_ precipitates at the entrance of the nanotubes [27,28]. We also find that the ScCO_2_ fluid can effectively remove these Ti(OH)_4_ precipitates from the nanotube surface, ultimately resulting in purer nanotube topography for these nanotubes (see Figure 1e,f,g,h). This result shows that the ScCO_2_ treatment can be an effective approach for surface cleaning for Ti-based nanostructured implants.

**Figure 3:**
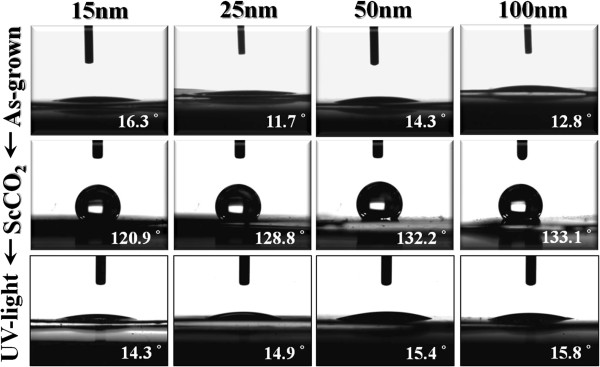
**Optical images showing water droplets.** On the as-grown (upper column), ScCO_2_-treated (middle column), and ScCO_2_-treated TiO_2_ nanotubes with UV light irradiation (lower column), respectively. Contact angles are denoted in the images.

An earlier work has shown that cell attachment, spreading, and cytoskeletal organization are significantly greater on hydrophilic surfaces relative to hydrophobic surfaces [29]. Das et al. further indicated that a low contact angle leads to high surface energy, which is also an important factor that contributes to better cell attachment [30]. As mentioned previously, the ScCO_2_ treatment may substantially modify the surface chemistry of TiO_2_ and possibly change the surface wettability accordingly. It is thus essential to understand the influence of the ScCO_2_ treatment on the nanotube wettability. As shown in Figure 3, all as-grown TiO_2_ nanotubes are highly hydrophilic since their contact angles are quite small. Nevertheless, after the ScCO_2_ treatment, these nanotube samples become hydrophobic. Once these ScCO_2_-treated TiO_2_ nanotubes were irradiated with UV light, their surface hydrophobicity transforms to high hydrophilicity again. These UV-irradiated TiO_2_ nanotubes could preserve their high hydrophilicity for at least 1 month. It should be noted that even with different nanotube diameters, all nanotube samples show similar behavior in the transition of surface wettability. There are two equations in the literature that describe the water contact angle on rough surfaces. One is Wenzel's law, which describes the small contact angle on hydrophilic materials [31]. The other one is formulated by Cassie and Baxter [32], which is generally valid for heterogeneous surfaces composed of air and a solid with hydrophobicity. Both models discuss the surface wettability based on the surface roughness and geometry of materials. Our results indicate that in these TiO_2_ nanotubes of different diameters (i.e., with different geometric factors), surface chemistry effects prevail in their surface wettability behavior.

**Figure 4:**
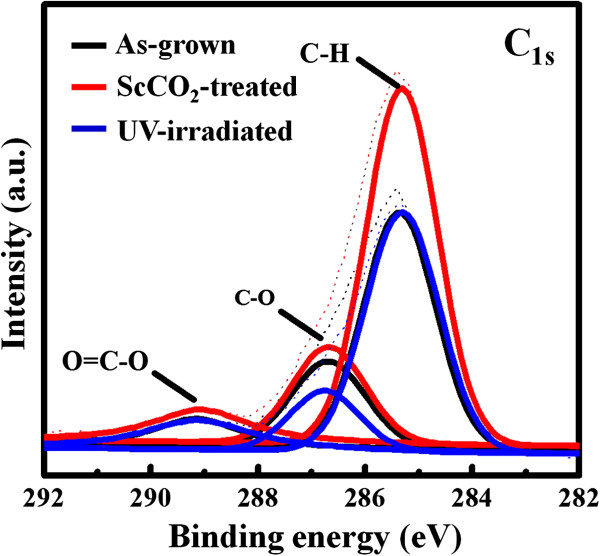
**XPS surface analysis results, in terms of spectra for C 1 *s *.** Of the as-grown, ScCO_2_-treated, and ScCO_2_-treated TiO_2_ nanotubes of 100 nm in diameter with UV light irradiation.

**Figure 5:**
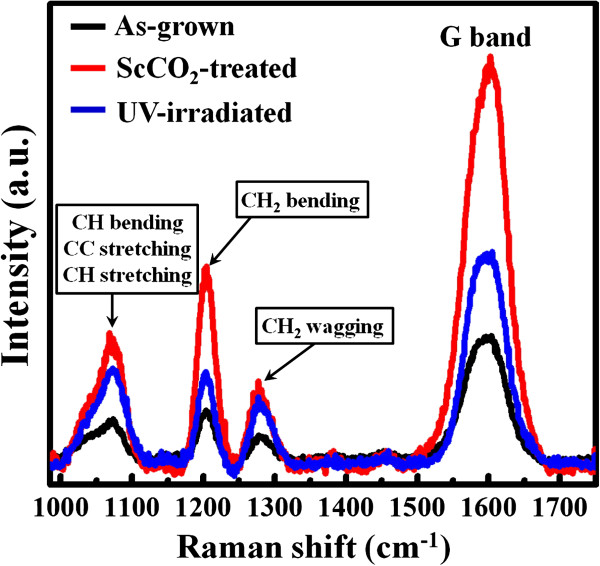
**Raman spectra of as-grown 100-nm-diameter TiO**_**2 **_**nanotubes treated with ScCO**_**2 **_**fluid and subsequent UV light irradiation.**

We attempt to elaborate the possible mechanism for the observed transitions in wettability in this study. First, we can almost exclude the possibility that the absorption of non-polar CO_2_ molecules on the nanotube surface leads to the hydrophobicity by the fact that the ScCO_2_-treated nanotubes still remain hydrophobic when kept in the atmosphere for more than 1 month. Another possibility is that newly forming functional groups on the nanotube surface during the ScCO_2_ process change the surface chemistry and wettability. Figure 4 shows the XPS surface analysis results, in terms of the C 1*s* spectra, of the as-grown, ScCO_2_-treated, and ScCO_2_-treated TiO_2_ nanotubes of 100 nm in diameter with UV light irradiation, respectively. We find that the C-H signal in the as-grown sample becomes much stronger (more significantly than other signals) after the ScCO_2_ treatment. It suggests that numerous C-H functional groups form on the TiO_2_ nanotube surface, possibly resulting from the reaction between the ScCO_2_ and TiO_2_·*x*H_2_O or Ti(OH)_4_. It has been reported that the C-H functional groups are non-polar with a hydrophobic nature [33]. This can explain why the TiO_2_ nanotubes become hydrophobic after the ScCO_2_ treatment. In addition, it is well known that TiO_2_ can act as a photocatalyst under UV light irradiation [34]. The C-H functional groups can be effectively photo-oxidized on the TiO_2_ nanotubes under UV light irradiation [35]. Therefore, the ScCO_2_-treated nanotubes recover their surface wettability after being irradiated with the UV light. This also agrees with the XPS result that C-H signal diminishes in the UV light-irradiated sample. The Raman spectra in Figure 5 show a similar trend. The carbon-related Raman vibrations in the as-grown sample, including C-H bending, C-H stretching, and H-C-H bending modes [36,37], become significantly stronger after the ScCO_2_ treatment and then diminish under UV light irradiation, indicating that the C-H functional groups indeed form on the nanotube surface and then are being photo-oxidized under UV light exposure. In addition, we find that almost no carbon-related Raman signals can be seen for the annealed TiO_2_ nanotubes before and after the ScCO_2_ treatment. This observation also supports our inference that the forming C-H functional groups may result from the reaction between ScCO_2_ and TiO_2_·*x*H_2_O or Ti(OH)_4_ nanotube surface. Since the annealed nanotubes have been dehydrated and transformed into a pure anatase phase, the reaction between ScCO_2_ and TiO_2_·*x*H_2_O or Ti(OH)_4_ to generate the C-H functional groups does not occur during the process.

**Figure 6:**
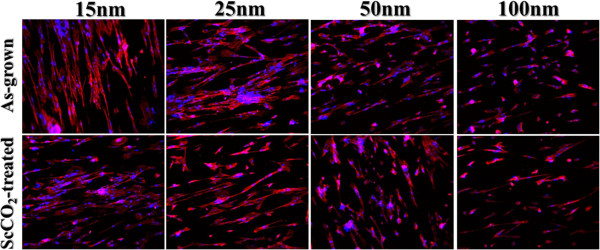
**Fluorescent images of the fibroblast cell attachment.** On the as-grown (upper column) and ScCO_2_-treated (lower column) TiO_2_ nanotubes of different diameters. The red fluorescence indicates cytoskeletal protein actin filament, and the blue fluorescence indicates nuclei.

**Figure 7:**
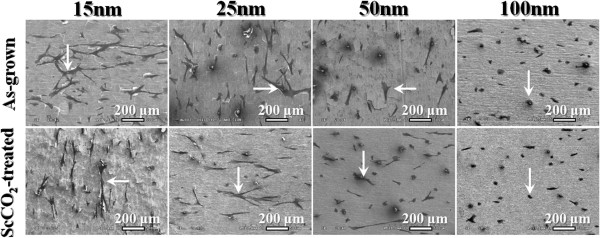
**SEM images showing the cell adhesion and proliferation of human fibroblast cells.** On the as-grown (upper column) and ScCO_2_-treated (lower column) TiO_2_ nanotubes of different diameters.

The human fibroblast cell behavior in response to the as-grown and ScCO_2_-treated TiO_2_ nanotubes is studied. To evaluate the fibroblast cell attachment on the TiO_2_ nanotubes, cytoskeleton actin was stained with rhodamine phalloidin that expressed red fluorescence and nuclei were stained with DAPI that expressed blue fluorescence. The actin immunostaining shows very different cell-material contact morphology for the TiO_2_ nanotubes of different diameters (see Figure 6). For both as-grown and ScCO_2_-treated samples, there are much longer and well-defined actin fibers noted on fibroblasts cultured on 25-nm- and smaller diameter nanotubes with respect to the larger ones. It is well known that cells have to adhere on a material surface first and then spread for further cell division. Better cell adhesion can cause more activation of intracellular signaling cascades through integrin coupled to actin cytoskeleton [38,39]. Therefore, the smaller diameter nanotubes give more focal points for fibroblasts to get attached, thus help in the cell adhesion. FE-SEM was used for the detailed observation of cell adhesion (see Figure 7). The fibroblasts on the smaller diameter TiO_2_ nanotubes reveal good cell adhesion with an elongated flatten morphology, while those on the 50-nm- and larger diameter nanotubes show rounded morphology and lack of cell spreading. It is known that cells recognize surface features when a suitable site for adhesion has been detected. Cells then stabilize the contact by forming focal adhesions and mature actin fibers, followed by recruiting tubulin microtubules [38]. The actin cytoskeleton is linked to integrins which are located within the adhesions. Our findings suggest that the cytoskeleton on the smaller diameter nanotubes should be formed better than that on the larger diameter ones for both as-grown and ScCO_2_-treated nanotubes. These observations also indicate that with UV light irradiation to recover the surface wettability, ScCO_2_-treated TiO_2_ nanotube surface is suitable for the cell adhesion.

**Figure 8:**
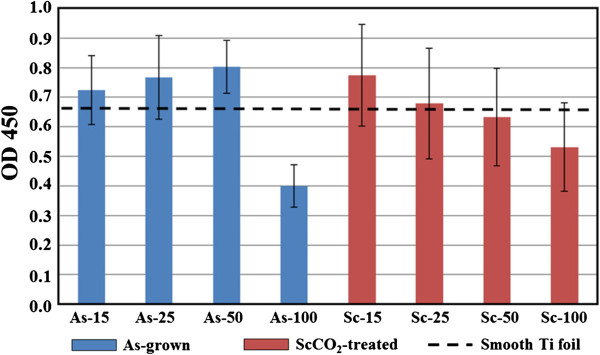
**Optical densities (QD) measured after the culture of human fibroblast cells.** On the as-grown and ScCO_2_-treated TiO_2_ nanotubes of different diameters.

The WST-1 assay was employed for further evaluating the fibroblast cell proliferation on the as-grown and ScCO_2_-treated TiO_2_ nanotubes of different diameters. Figure 8 shows the comparison of optical densities measured from the WST-1 assay results. We find that cell proliferation is lowest for the largest diameter of 100 nm in both as-grown and ScCO_2_-treated TiO_2_ nanotube samples. In addition, the ScCO_2_-treated TiO_2_ nanotubes appear to exhibit a monotonically increasing trend in cell proliferation with decreasing nanotube diameter. This trend is not so obvious in the as-grown samples. It indicates that human fibroblast cells show more obvious diameter-specific behavior on the ScCO_2_-treated TiO_2_ nanotubes than on the as-grown ones. As discussed previously, the ScCO_2_ fluid can effectively remove the disordered Ti(OH)_4_ precipitates from the nanotube surface. This may result in purer nanotube topography and thus more sensitive cell response to the diameter of the ScCO_2_-treated nanotubes. Eventually, for the smallest diameter of 15 nm, ScCO_2_-treated TiO_2_ nanotubes reveal higher biocompatibility than the as-grown sample.

## Conclusions

In conclusion, this study investigates the diameter-sensitive biocompatibility of ScCO_2_-treated TiO_2_ nanotubes of different diameters prepared by electrochemical anodization. We find that ScCO_2_-treated TiO_2_ nanotubes can effectively recover their surface wettability under UV light irradiation as a result of photo-oxidation of C-H functional groups formed on the surface. It is demonstrated that human fibroblast cells show more obvious diameter-specific behavior on the ScCO_2_-treated nanotubes than on the as-grown ones, which can be attributed to the removal of disordered Ti(OH)_4_ precipitates from the nanotube surface by the ScCO_2_ fluid. This results in purer nanotube topography, stronger diameter dependence of cell activity, and thus higher biocompatibility for the 15-nm-diameter ScCO_2_-treated TiO_2_ nanotubes than the as-grown sample. This study demonstrates that the use of ScCO_2_ fluid can be an effective, appropriate, and promising approach for surface treatments or modifications of bio-implants.

## Competing interests

The authors declare that they have no competing interests.

## Authors’ contributions

MYL conducted the *in vitro* experiments and drafted that part of the manuscript. CPL prepared all nanotube samples and analyzed their surface wettability. HHH revised the manuscript. JKC conducted the ScCO_2_ experiments and XPS analysis. SWL designed the study and wrote the manuscript. All authors read and approved the final manuscript.

## Authors’ information

MYL is currently a visiting staff of the Department of Otolaryngology at Taipei Veterans General Hospital and also a Ph.D. candidate of National Yang-Ming University (Taiwan). CPL is currently a Master's degree student of National Central University (Taiwan). HHH is a professor of the Department of Dentistry at National Yang-Ming University (Taiwan). JKC is an assistant professor of the Institute of Materials Science and Engineering at National Central University (Taiwan). SWL is an associate professor of the Institute of Materials Science and Engineering at National Central University (Taiwan).
